# Focused ultrasound development and clinical adoption: 2013 update on the growth of the field

**DOI:** 10.1186/2050-5736-2-2

**Published:** 2014-02-27

**Authors:** Dasha Tyshlek, Jean-Francois Aubry, Gail ter Haar, Arik Hananel, Jessica Foley, Matthew Eames, Neal Kassell, Heather Huff Simonin

**Affiliations:** 1 Focused Ultrasound Foundation, Charlottesville, VA 22903, USA; 2 Department of Radiation Oncology, University of Virginia, Charlottesville, VA 22901, USA; 3 Institut Langevin, CNRS UMR 7587, ESPCI ParisTech, INSERM U979, Paris 75005, France; 4 Division of Radiotherapy and Imaging, The Institute of Cancer Research, Royal Marsden Hospital, Sutton, Surrey, UK; 5 Department of Neurosurgery, University of Virginia, Charlottesville, VA 22901, USA

## Abstract

The field of therapeutic focused ultrasound, which first emerged in the 1940s, has seen significant growth, particularly over the past decade. The eventual widespread clinical adoption of this non-invasive therapeutic modality require continued progress, in a multitude of activities including technical, pre-clinical, and clinical research, regulatory approval and reimbursement, manufacturer growth, and other commercial and public sector investments into the field, all within a multi-stakeholder environment. We present here a snapshot of the field of focused ultrasound and describe how it has progressed over the past several decades. It is assessed using metrics which include quantity and breadth of academic work (presentations, publications), funding trends, manufacturer presence in the field, number of treated patients, number of indications reaching first-in-human status, and quantity and breadth of clinical indications.

## Content

### Introduction

The first publication to demonstrate focused ultrasound's potential therapeutic use appeared in 1942 [1]. Over the following decade, focused ultrasound was investigated as a potential treatment for neurofunctional disorders, specifically Parkinson's disease [2,3]. At this initial stage, the transmission of acoustic energy into the brain was an invasive procedure because of the reflection, absorption, and diffraction produced by the skull. Real-time image guidance was not available at the time. An offline X-ray was therefore used to guide the beams from the single-element therapeutic ultrasound transducers which were mounted on a stereotactic frame.

The Food and Drug Administration (FDA) first approved a focused ultrasound (FUS) device in 1988 [4,5]. This device provided non-invasive treatment of glaucoma under ultrasound and optical imaging guidance [6-8].

In the 1950s, Vallancien et al. reported clinical results with a new system that employed ultrasound imaging for guidance and a thermocouple for thermal measurements. This system was used to treat patients with bladder cancer [9] and prostatic, liver, and renal tumors [10]. This led in 2000 to the first Conformité Européenne (CE) approval for this indication.

Magnetic resonance imaging (MRI)-guided focused ultrasound devices were introduced in the early 1990s [11-14], with the first device obtaining CE approval in 2003 and FDA approval in 2004, for the treatment of symptomatic uterine fibroids.

The development of multielement transducers [15,16] allowed electronic beam steering around the geometrical focus [17,18] and the production of multiple simultaneous foci [19]. Electronic beam steering can be used either to treat large areas without moving the therapeutic probe [20] or to perform motion compensation [21]. The phase of the signal at each element can also be adjusted to provide focusing through aberrating structures such as the skull [22-27] or the ribs [28-32].

To date, a significant amount of work has been performed using various ultrasound (US)-guided and MRI-guided systems for the treatment of symptomatic uterine fibroids [33,34]; brain tumors [35-37]; painful bone metastases [38,39]; prostatic [40-42], pancreatic [43], and breast [44] cancer; and abdominal tumors [45-47].

To date, more than 80,000 patients have been treated globally using a variety of ultrasound-guided and MRI-guided therapeutic high-intensity focused ultrasound devices.

### Purpose

In order to evaluate the growth of the field of focused ultrasound, and to shine a spotlight on its current level of development and clinical adoption, two primary areas have been assessed, namely historical progress in financial support and general awareness of focused ultrasound, and the health impacts of the field.

The financial support and general awareness of focused ultrasound can be assessed by tracking the amount of research funding provided to focused ultrasound projects, the number of publications in peer-reviewed journals, and general awareness metrics such as visits to specific focused ultrasound websites, including its Wikipedia page.

The impact of focused ultrasound on the health of the global community can be gleaned through examination of the number of clinical indications reaching first-in-human stage and of how many patients have been treated.

### Methods

For this evaluation, several metrics and data sources were used including the annual number of publications (Medline) and citations (Thompson Reuters Web of Science); the number of abstracts presented in meetings dedicated to focused ultrasound (the annual meeting of the International Society of Therapeutic Ultrasound (ISTU—founded in 2001), the biennial meeting organized by the Focused Ultrasound Foundation (FUSF—founded in 2006), and the European Focused Ultrasound Working Group (EFUS—founded in 2011)); the clinical indications that have reached the level of human feasibility trials (obtained from literature review and communications with relevant researchers); the number of annual treatments administered (from manufacturers' reports); manufacturer growth within the field; and the amount of National Institutes of Health (NIH) funding allocated to focused ultrasound research. In addition, the number of hits on the FUS Foundation website and FUS Wikipedia page were used as indicators for general awareness of and interest in the field. This data was used to evaluate the historical progress and current status of FUS as a treatment modality for a variety of clinical indications.

The annual number of publications was obtained using the Medline trends online tool, with the search term ‘focused ultrasound’ which is inclusive for most other terms used in this field. The Thomson Reuters Web of Science was used to assess the number of citations per year, with the same search criterion.

Organizers of focused ultrasound-specific scientific meetings were contacted in order to assess the number of abstracts presented at these meetings. Where these organizers could not be reached, the number of publications listed in the Proceedings for these conferences was used.

Manufacturer count and type were obtained by requesting information from industry executives, online research, and information collected through focused ultrasound symposia registration and sponsorship data. Only manufacturers developing an image-guided, non-invasive device using focused ultrasound for therapy or pre-clinical research, including systems solely for animal research, were included in these metrics.

The number of patients treated was acquired for each clinical indication by contacting the relevant device manufacturers. To identify the time point at which each clinical indication reached a feasibility clinical trial, the authors surveyed researchers in the relevant clinical field, reviewed articles, and conducted specific literature searches.

NIH funding allocation to focused ultrasound research was obtained using the NIH RePorter database. The terms ‘high intensity focused ultrasound,’ ‘focused ultrasound therapy,’ ‘ablation,’ ‘drug delivery,’ and ‘HIFU’ were searched for separately, but the data was aggregated and duplicates were eliminated. Search results were further refined based on the relevance of the listed projects. Data were analyzed and presented by year, total HIFU annual funding, and funding as a percent of the total NIH budget. Total annual NIH research spending was extracted from NIH annual reports. In this work we present only NIH funding and not funding from sources in Europe and Asia because these data were not publicly available in a single location. We assume the NIH funding could be considered as a surrogate for government funding worldwide.

The visits to the FUS Foundation website were analyzed using Listrack and Google analytics. The number of Wikipedia page views was collected using stats.grok.se with the key word ‘high-intensity focused ultrasound.’ These general awareness metrics data were analyzed and are presented by month or quarter, with significant events highlighted on the timeline.

The data collected and the search terms apply to therapeutic focused ultrasound, with the majority on it being for high-intensity focused ultrasound, and thus distinct from non-focused therapeutic ultrasound that is used for physiotherapy or other indications.

### Results

**Figure 1:**
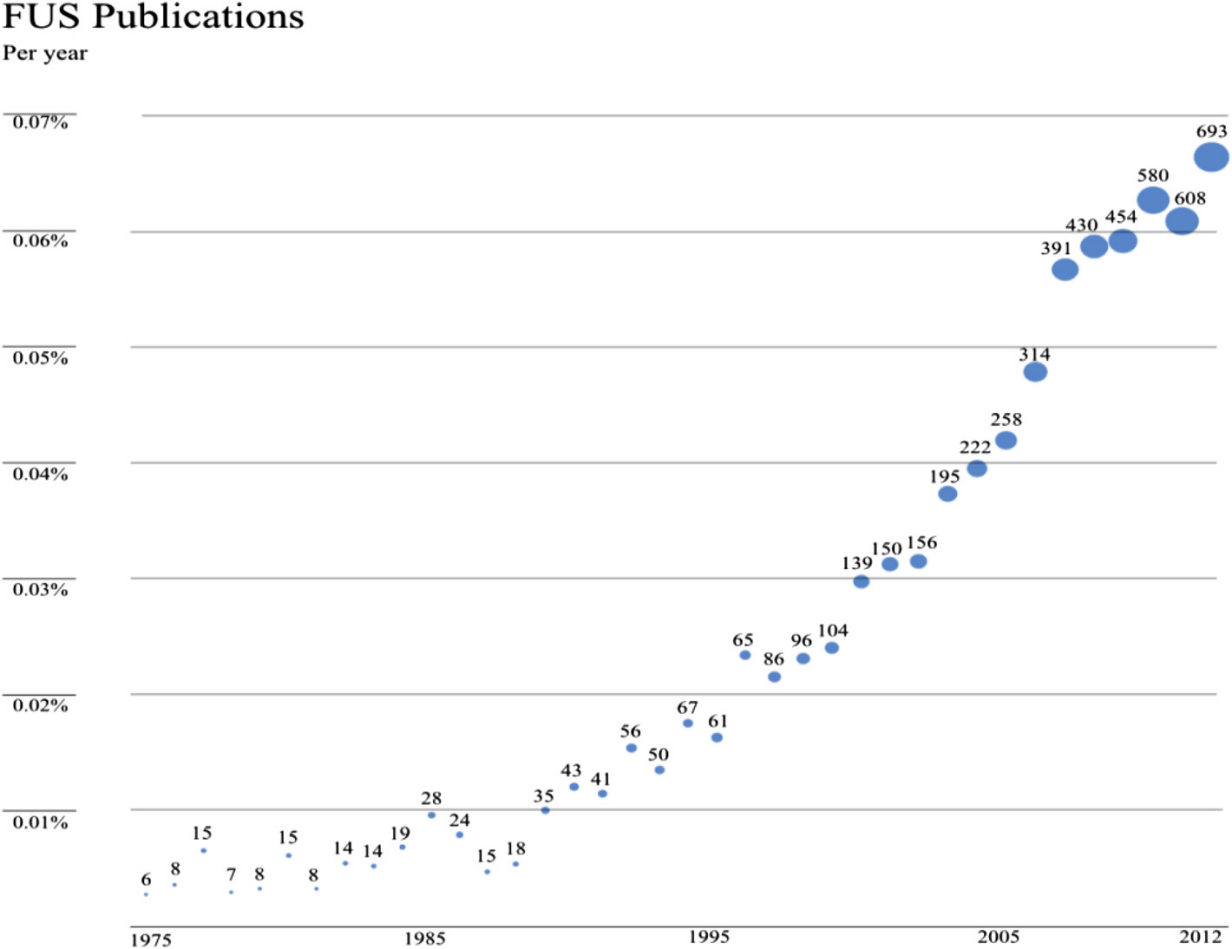
Focused ultrasound publications: annual number and percentage of overall of publications in Medline.

**Figure 2:**
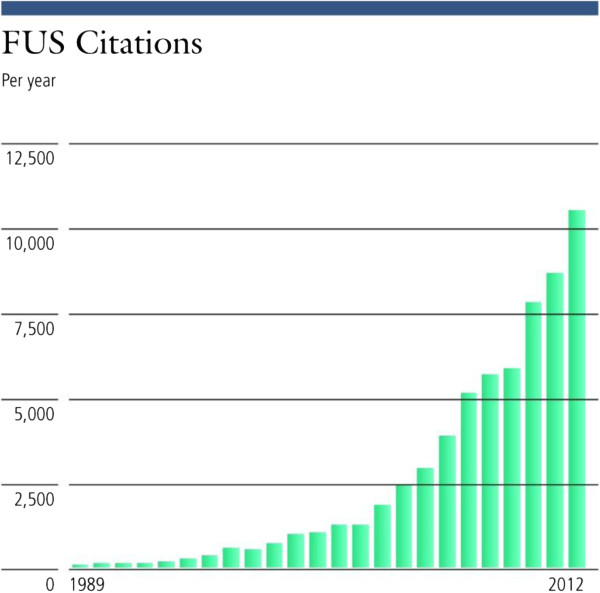
Annual number of citations of focused ultrasound publications.

As shown in Figures 1 and 2, the absolute number of publications and citations has been increasing since the beginning of the 1990s as has the ratio of publications on focused ultrasound to the overall number of publications in Medline.

**Figure 3:**
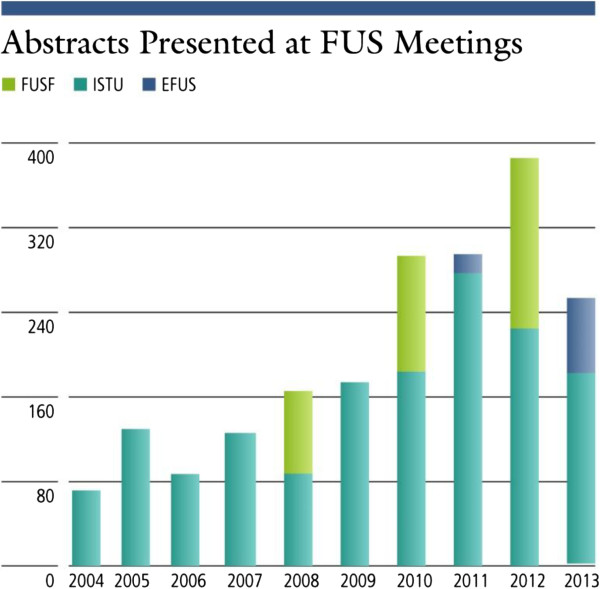
Number of abstracts presented at various focused ultrasound centric meetings: ISTU, EFUS, and FUS Foundation meetings.

**Figure 4:**
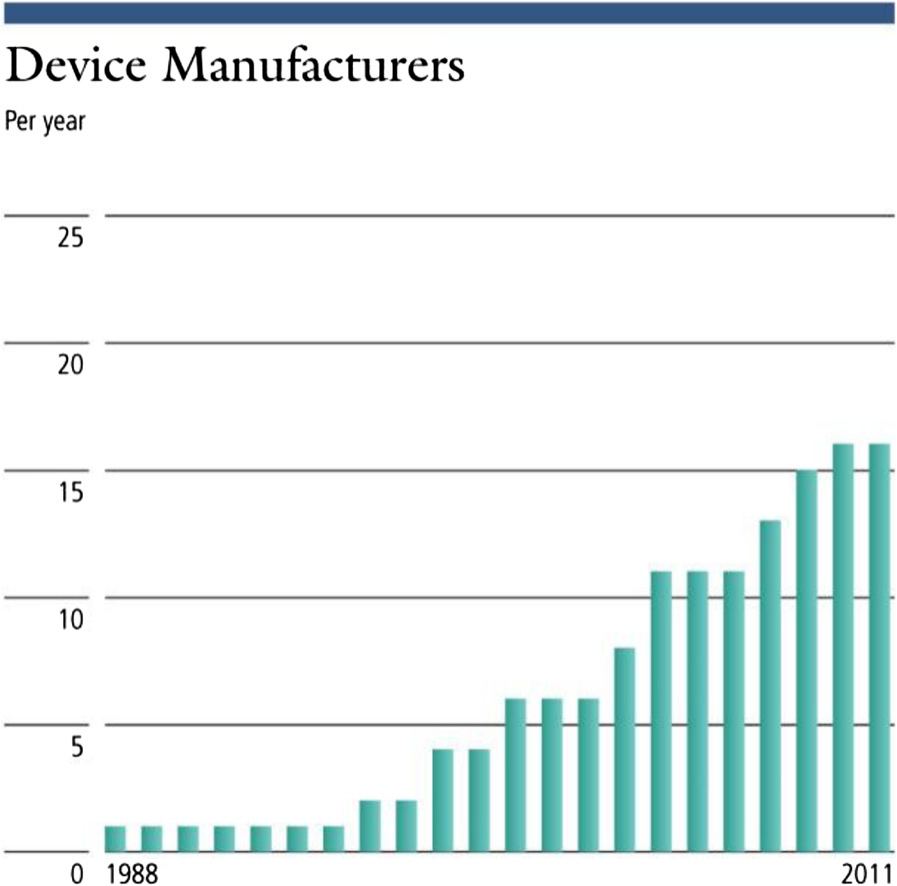
Number of FUS device manufacturers by year of establishment or entrance into the field of FUS.

Figure 3 indicates an increase in the number of abstracts presented in FUS centric meetings. A similar trend of growth is seen in the number of manufacturers who are developing and selling devices for both clinical and animal research using various guidance methods as is shown in Figure 4 and Table 1.

**Table 1:** **Focused ultrasound device manufacturers**

**Company**	**Founded**	**Guidance and usage**
EDAP TMS	1979	US
SonaCare Medical (previously US-HIFU, International HIFU and Focus Surgery)	1997	US
Chongqing HAIFU	1999	Both
China Medical	1999	US
Insightec	1999	MR
Image Guided Therapy	2001	MR—animals
Shanghai A&S	2001	Both
Mirabilis	2004	US
Theraclion	2004	US
Medsonic	2005	MR
Philips Healthcare	2005	MR
Supersonic Imagine	2005	Both
Profound	2008	MR
EyeTechCare	2008	Visual
Alpinion	2008	US
International Cardio Corporation	2009	US
Kona Medical	2009	US
Histosonic	2009	US
FUS instruments	2009	MR—animals
Acublate	2010	Both

Criteria include guidance method (US, MR, other) and usage (if used only for pre-clinical studies, this is marked as ‘animal’).

**Figure 5:**
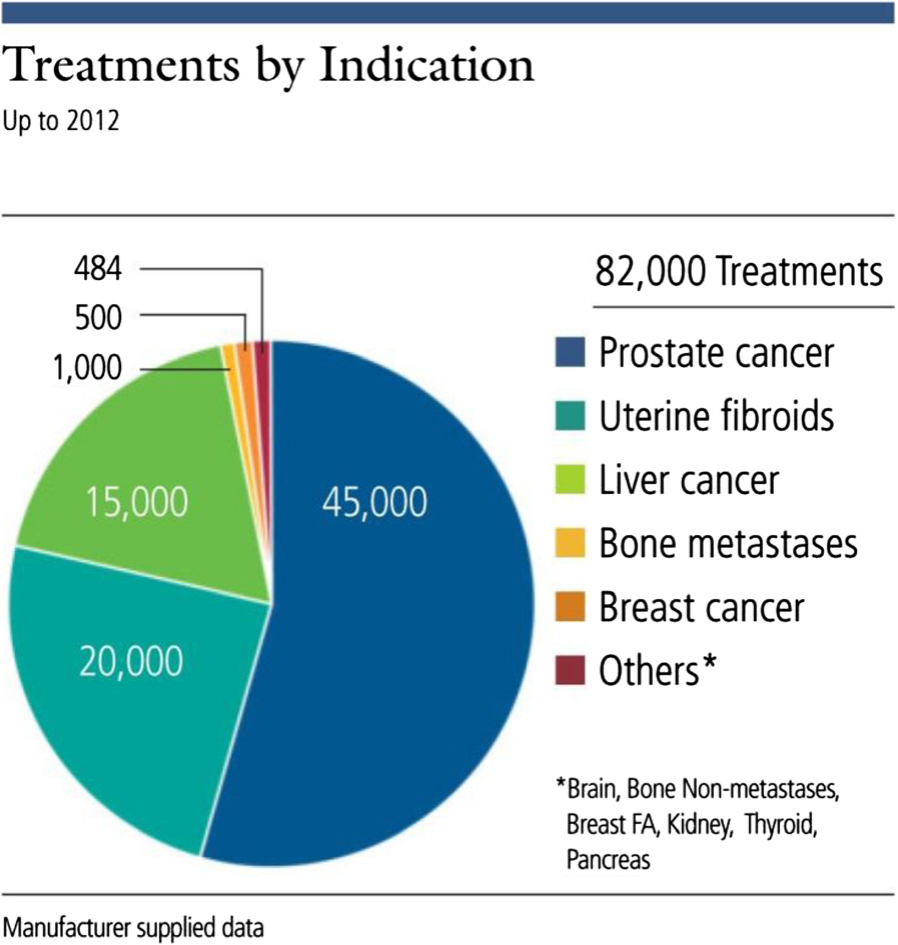
Number of patients treated by focused ultrasound for various clinical indications.

**Figure 6:**
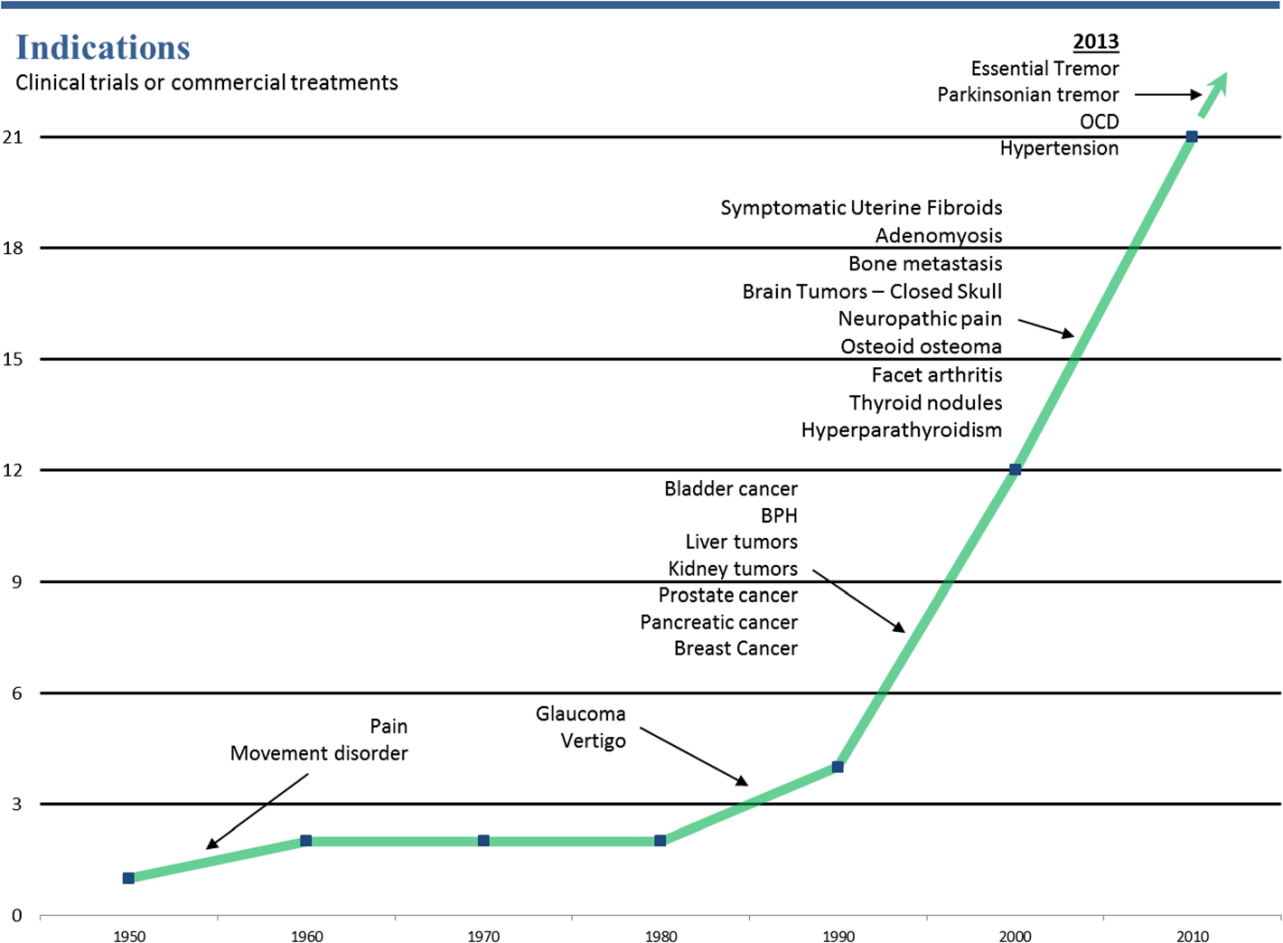
Number of indications reaching first-in-human over time.

**Figure 7:**
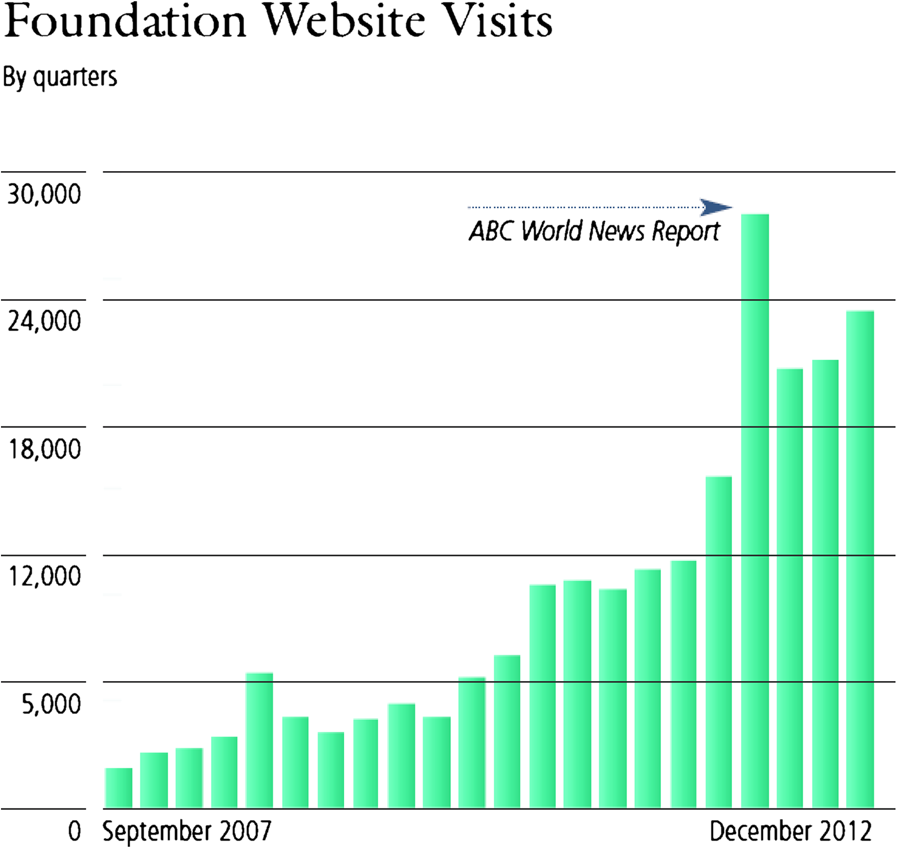
Number of visits per quarter to the FUS Foundation website.

**Figure 8:**
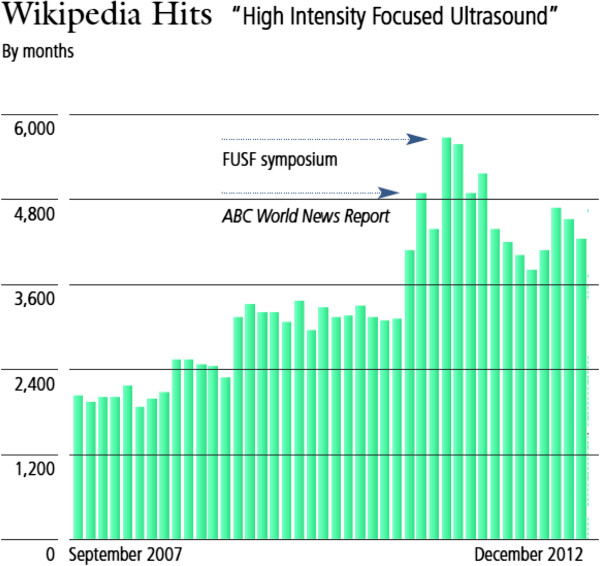
Number of FUS-specific Wikipedia searches.

**Figure 9:**
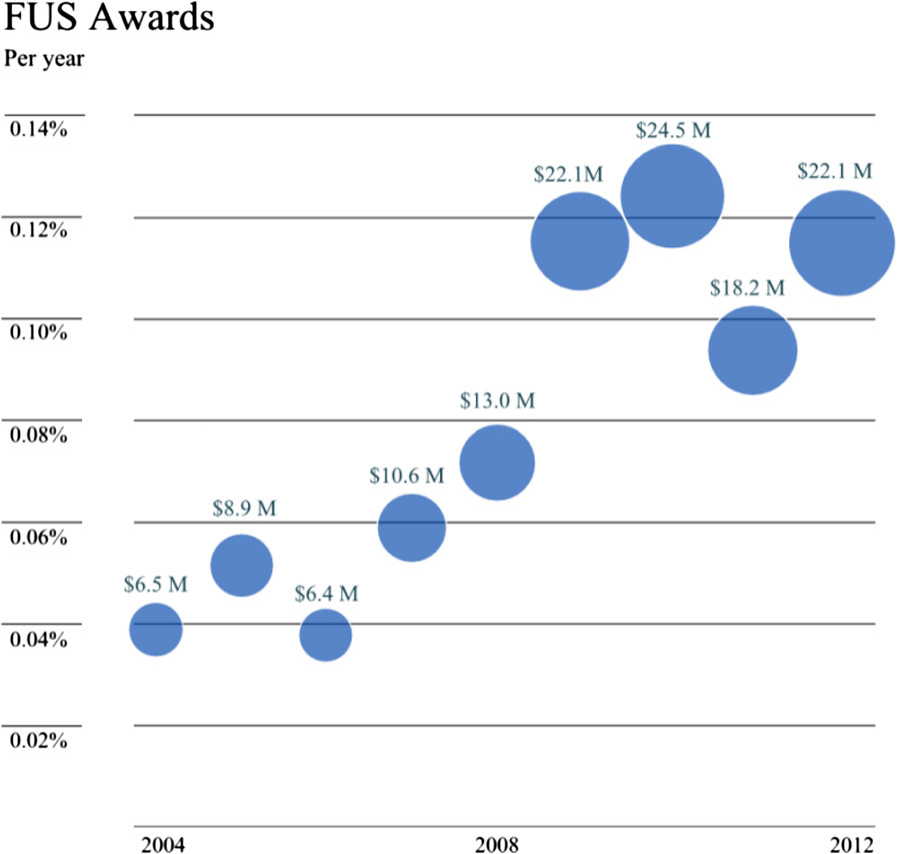
NIH funding for focused ultrasound projects and percentage of FUS research funding in the NIH budget.

As shown in Figure 5, more than 80,000 patients have been treated using FUS for multiple clinical indications. Many more indications are actively being researched and tested as shown in Figure 6, which demonstrates graphically the date of the first-in-human focused ultrasound treatment for each indication. Traffic to the FUS Foundation website and to the Wikipedia article on focused ultrasound has been increasing, as can be seen in Figures 7 and 8, indicating growth in the awareness of ultrasound. On these two figures, we have also marked the date of the most recent FUS Foundation symposium and recent news coverage in ABC, which created a local peak in the graphs. Additionally, Figure 9 shows that in the USA, policymaker awareness has also increased, as FUS has been receiving increased funding and has been growing in funding relative to total NIH finding.

### Discussion

The data presented shows evidence of progress in the field of focused ultrasound. This is demonstrated by the number of clinical indications explored, which has increased from 1 to 21 since 1950, or by the increase in the amount of research as indicated by the steady increase in the number of publications. In addition, the number of abstracts at focused ultrasound events and symposia is also on the rise, indicating a higher level of activity (a similar trend may exist in non-focused ultrasound-specific symposia but was more challenging to measure). Publications and citations for focused ultrasound have also been increasing yearly and by a greater percentage than the total number of medical publications, indicating that focused ultrasound research is growing faster than medical research overall. A similar trend of growth could be seen also in the increase in funding allocated to focused ultrasound and by web traffic in sites dedicated to this topic.

This data, however, continues to pose important questions about metrics and data collection. How can the community measure progress in a field that is developing globally across many different clinical indications? Should progress be measured using translation and application or technological capability metrics? How can public awareness be measured and correlated with adoption?

Collection of relevant data remains a challenge for the focused ultrasound community. Most funding data is publicly available only for programs in the USA. This means that some global impacts cannot, as yet, be measured or compared. Additionally, research site, manufacturer, and clinical site information is collected only for the organizations that actively participate in the focused ultrasound community and regularly submit their information on a voluntary basis. This means that it is harder to collect data from some regions than others, skewing the geographic representations of the focused ultrasound field. The Focused Ultrasound Foundation would like to invite the community to engage with its business development team to enable better data collection and increased global awareness.

### Conclusion

The field of therapeutic focused ultrasound has been steadily growing since its inception in the 1940s. The results presented here, obtained using quantitative metrics and publically accessible data sources, offer a snapshot of the progress in the development and clinical adoption of this technology. Data indicates that there is widespread progress in scientific and clinical research which may lead to increased adoption of the technologies for the benefit of patients worldwide.

This data, however, continues to pose important questions, particularly for the Focused Ultrasound Foundation. How can future growth potential be identified and our resources mobilized to maximize progress in this area? How can we overcome the barriers along the pathway from idea conception to successful patient treatment, to catalyze the process and bring this non-invasive therapy to patients faster?

The Focused Ultrasound Foundation may be the most appropriate organization to continue to track the field's progress. If so, it needs input and guidance from the focused ultrasound community to strengthen these efforts. Suggestions for further metrics relevant to the development of the field and the sharing of information in the areas currently being monitored would be welcomed.

The FUS Foundation aims to provide the most accurate information. If you have more current information, please send it to progress@fusfoundation.org.
